# Microbiota Alters Urinary Bladder Weight and Gene Expression

**DOI:** 10.3390/microorganisms8030421

**Published:** 2020-03-17

**Authors:** Blanka Roje, Anamaria Elek, Vinko Palada, Joana Bom, Aida Iljazović, Ana Šimić, Lana Sušak, Katarina Vilović, Till Strowig, Kristian Vlahoviček, Janoš Terzić

**Affiliations:** 1 Laboratory for Cancer Research, University of Split School of Medicine, 21000 Split, Croatia; blanka.roje@mefst.hr (B.R.); simic.ana1107@gmail.com (A.Š.); lana.susak.17@gmail.com (L.S.); 2 Bioinformatics Group, Division of Molecular Biology, Department of Biology, Faculty of Science, University of Zagreb, 10000 Zagreb, Croatia; anamariaelek@gmail.com (A.E.); kristian@bioinfo.hr (K.V.); 3 Department of Physiology and Pharmacology, Karolinska Institute, 17177 Solna, Sweden; vinko.palada@ki.se; 4 Instituto Gulbenkian de Ciência, 2784 Oeiras, Portugal; jbom@igc.gulbenkian.pt; 5 Helmholtz Institute for Infection Research, 38124 Braunschweig, Germany; Aida.Iljazovic@helmholtz-hzi.de (A.I.); Till.Strowig@helmholtz-hzi.de (T.S.); 6 Department of Pathology, University Hospital Split, 21000 Split, Croatia; kvilovic@mefst.hr; 7 School of Bioscience, University of Skövde, 54128 Skövde, Sweden

**Keywords:** germ-free, microbiome, urinary bladder, transcriptome, mouse

## Abstract

We studied the effect of microbiota on the transcriptome and weight of the urinary bladder by comparing germ-free (GF) and specific pathogen-free (SPF) housed mice. In total, 97 genes were differently expressed (fold change > ±2; false discovery rate (FDR) *p*-value < 0.01) between the groups, including genes regulating circadian rhythm (Per1, Per2 and Per3), extracellular matrix (Spo1, Spon2), and neuromuscular synaptic transmission (Slc18a3, Slc5a7, Chrnb4, Chrna3, Snap25). The highest increase in expression was observed for immunoglobulin genes (Igkv1-122, Igkv4-68) of unknown function, but surprisingly the absence of microbiota did not change the expression of the genes responsible for recognizing microbes and their products. We found that urinary bladder weight was approximately 25% lighter in GF mice (*p* = 0.09 for males, *p* = 0.005 for females) and in mice treated with broad spectrum of antibiotics (*p* = 0.0002). In conclusion, our data indicate that microbiota is an important determinant of urinary bladder physiology controlling its gene expression and size.

## 1. Introduction

The microbiome is an important determinant of our health. Sometimes, it is referred to as the “forgotten organ” because of its influence on various physiological processes and our wellbeing. Most microorganisms are found in the gastrointestinal tract and on the skin, but other body sites that act as barriers to the environment are also populated with microbes [1]. So far, the composition of microbiome has been linked to drug response, obesity, diabetes type 2, diet habits, and overall life span [1].

Contrary to the previous belief, the urinary bladder is not sterile. It contains a microbiome, which differs among individuals depending on age, sex, and health status [2]. The urethra represents a direct link between the bladder and the outside environment. With the permanent contact with urine as a permissive medium for bacterial growth, the urethra and urinary bladder are likely sites constantly challenged by microbial growth. However, the role of microbiota in the physiology and pathology of the urinary bladder is not well understood. The importance of the urinary bladder microbiome is nicely demonstrated by the modulation of the bladder microbiome by bacillus Calmette– Guérin (BCG), which is successfully used as a bladder cancer therapy [3]. Besides the urinary microbiome that can directly influence bladder through the urothelium or via metabolites discharged in the urine, the gut microbiota can indirectly influence remote parts of the body including urinary bladder physiology [1].

In the current study, we aimed to identify the effects of the microbiota on the bladder transcriptome by comparing germ-free (GF) mice with mice that harbour normal microbiota (i.e., specific pathogen-free, SPF) [4]. It has been previously reported that GF mice have decreased body fat and reduced metabolic rates [5]. In terms of anatomical differences, the livers of GF mice are smaller, the total surface of the small intestine decreased, while the caecum is larger [6]. Therefore, we also aimed to compare the tissue morphology and size of the urinary bladder between mice housed under GF conditions and their counterparts housed under standard SPF conditions. 

## 2. Materials and Methods

### 2.1. Animals

For gene expression analysis, we used 6 male and 6 female C57BL/6J GF mice (6-weeks-old), as well as the same number of their age and sex-matched specific-pathogen-free (SPF) counterparts (Jackson Laboratory, Bar Harbour, ME, USA). Animals were housed in the animal facility of Instituto Gulbenkian (IGC), Oeiras, Portugal, with 12-h light/12-h dark cycle, controlled temperature and humidity, and ad libitum access to water and food. The food (cat. no. SDS RM3-A-P, Dietex International, Essex, UK) and bedding for the GF mice were autoclaved and were not tested for LPS. The water was produced by reverse osmosis and UV treated before use and delivered sterile into the isolators. The GF mice were housed in rigid wall isolators with rapid transfer/DPTE doors (Getinge, Gothenburg, Sweden). All isolators followed a health monitoring program including a monthly microbiological screening. SPF mice were kept in individually ventilated cages. 

For the experiment with antibiotics, we used 10-week-old C56BL/6J male mice from the animal facility of University of Split School of Medicine (USSM), Split, Croatia. Mice were housed with the same conditions as the SPF mice from the IGC and were randomly assigned to treated (*n* = 13) and control group (*n* = 10). The treated group received an antibiotic mix in their drinking water, consisting of 100 μg mL^−1^ neomycin, 100 μg mL^−1^ metronidazole, 50 μg mL^−1^ streptomycin and 100 U mL^−1^ penicillin (all from Sigma-Aldrich, St Louis, MO, USA) and 50 μg mL^−1^ vancomycin (Pharma Swiss, Belgrade, Serbia) and were supplied fresh antibiotics every week for four weeks, as previously described [7]. Control mice were given autoclaved water. 

Finally, for the estimation of bladder size five GF and four SPF male mice (substrain C56BL/6N) from Helmholtz Centre for Infection (HZI) Research in Braunschweig, Germany, aged 9 weeks were used. SPF mice were held in individually ventilated cages (IVC) and germ-free mice were kept in isolator cages under sterile conditions (Getinge). Water and lighting conditions were as mentioned above. The food (cat. No. V1124-300, ssniff-Spezialdiäten GmbH, Soest, Germany) was sterilized by gamma-irradiation with 50kGy and was not tested for LPS. Upon harvesting, bladder size was measured by a calliper and bladders were weighed on an analytical scale.

Animal work was approved by the Ministry of Agriculture of the Republic of Croatia Permit number 525-10/0255-15-5. 

### 2.2. Tissue Collection and Histology

Right after harvesting, the remaining urine was absorbed from the bladders using tissue paper and bladders were weighed on an analytical scale. Tissues were then cut medially into approximately two equal halves using a scalpel. One half was immediately immersed in 10% neutral buffered formalin for 24 h while the other half was first snap frozen and then stored in liquid nitrogen for later use. After fixation, tissues were dehydrated using a series of ethanol dilutions, cleared from ethanol in three series of xylene, cleared of xylene in the first paraffin, and embedded in the second paraffin. Embedded tissues were then cut into 5-µm sections and stained with hematoxylin and eosin for microscopical examination. Histological assessment was performed by a trained pathologist.

### 2.3. RNA Isolation 

RNA was isolated from the frozen bladder specimens using Qiazol reagent (Qiagen, Hilden, Germany). The quantity and purity of RNA samples were determined using 260/280 and 260/230 ratios by Nanodrop 2000 (Thermofisher, Waltham, MA, USA). The 260/280 ratios were from 1.8 to 2, while 260/230 ratios were from 1–1.9. RNA integrity and potential DNA contamination were analyzed by agarose gel electrophoresis. Additionally, the RNA integrity of samples used for RNA-seq was determined by Agilent 2100 chips (Agilent Technologies, Inc., Santa Clara, CA, USA). All RNA samples had RIN >8. Electrophoresis confirmed that RNA samples did not have significant contamination with genomic DNA. 

### 2.4. RNA-Sequencing

The samples in the RNA Seq analysis consisted of pooled RNA from bladders of two male mice, using 1.2 micrograms of RNA from each animal. In total, 3 pooled samples per group were used. The cDNA library preparation and RNA-sequencing were done at the Novogene, Beijing, China. The mRNA was purified from total RNA using poly-T oligo-attached magnetic beads and the paired-end sequencing was performed by Illumina HiSeq2000 (Illumina, San Diego, CA, USA).

The average length of the reads was 150bp and 40M paired reads were generated per sample. 

### 2.5. RNA-Sequencing Data Analysis

Mouse reference genome mm10 was used to map raw sequencing reads from FASTQ files. Output in BAM file format was further analysed by Cufflinks and Cuffdiff (ver. 0.12.1) to calculate the abundance of transcripts and differential gene expression using FDR < 0.01 as a cut-off value using R programming language (ver. 3.5.0) [8]. The abundance of gene transcripts was expressed in RPKM (reads per kilobase of transcript). DESeq2 (ver. 1.22.1) package was used for differential gene expression analysis using a matrix with counted reads mapped to each individual gene [9]. The total number of reads per sample and number of mapped reads can be seen in Table S1. Differential gene expression and gene-set enrichment analysis with Kyoto Encyclopedia of Genes and Genomes (KEGG) pathways were performed [10,11]. R package gage was used for generally applicable gene-set enrichment (GAGE, ver. 2.32.0) analysis to identify non-redundant significantly altered pathways in GF versus SPF samples [12]. Pathview package (ver. 1.22.0) was used to visualize maps of the KEGG pathways [13]. The colouring is rendered from the summary log_2_ (fold change) of all genes in one gene node, representing multiple genes/proteins with a redundant functional role. Gene ontology (GO) analysis was done using PANTHER Overrepresentation Test (Released 20171205). PCA component analysis was done in R, on rlog transformed read counts.

### 2.6. Quantitative PCR

Quantitative PCR (qPCR) analysis of expression of 13 exemplar genes was performed in order to confirm the results of RNA-sequencing. One microgram of RNA was isolated from the mice bladders and reverse transcribed into cDNA with Ambion Reverse Transcription Kit (Thermo Fisher Scientific, Waltham, MA, USA). The qPCR reaction was performed using PowerUp™ SYBR™ Green Master Mix (Thermo Fisher Scientific) at Applied Biosystems™ 7500 Real-Time PCR System (Thermo Fisher Scientific). The following conditions were used for qPCR reaction: 50 °C for 2 min, 95 °C for 10 min, followed by 40 cycles of amplification (95 °C for 15 s, 60 °C for 1 min with plate reading) and a melting curve analysis. The fold change of the expression of each gene was calculated by ΔΔCt method as following: ΔCt (calculated as Ct(GOI)–Ct(Actb)) of each GF sample was subtracted from the average ΔCt of all SPF samples to obtain ΔΔCt, and the mean of −ΔΔCt of all GF samples was used as the measure of fold change of gene expression [14]. All reactions were performed in duplicates. Each run included Actb as a reference gene control. Reactions were done on samples from both male and female bladders. All primers were designed using online Primer-BLAST tool to span exon-exon junctions and align to all gene isoform transcripts (Table 1). The primer efficiency was assessed by four 5-fold dilutions starting from 4x concentrated cDNA and primers with efficiency above 90% were used. Several reference genes were checked, and Actin B was most stably expressed in all samples and therefore used in ∆∆Ct calculations.

### 2.7. Statistical Analysis

Statistical analysis of RNA-seq data was performed using DESeq2 with recommended default parameters. In short, this includes fitting a negative binomial generalized linear model to read counts, normalization by size factors and dispersion estimates, and testing for significance using the Wald test. FDR < 0.01 was used to determine differentially expressed genes between GF and SPF mice. A fold change of 2 or greater was taken as cut-off value. Fisher’s exact with FDR multiple test correction was used for GO analysis with a cut-off FDR < 0.05.

RNA-seq and qPCR correlation was assessed using Pearson correlation coefficient and its significance using two-tailed T-test. For the analysis of bladder weight, bladder-to-body weight ratio and bladder size, Shapiro-Wilk test was performed to check normality of the data (Supplementary Table S6). Significance was determined by two-tailed Student T-test for normally distributed data and Mann-Whitney rank test for the skewed data. Graphs were done in GraphPad Prism 8.2.1 for Windows (GraphPad Software, La Jolla, CA, USA).

## 3. Results

### 3.1. Organ and Tissue Assessment

**Figure 1:**
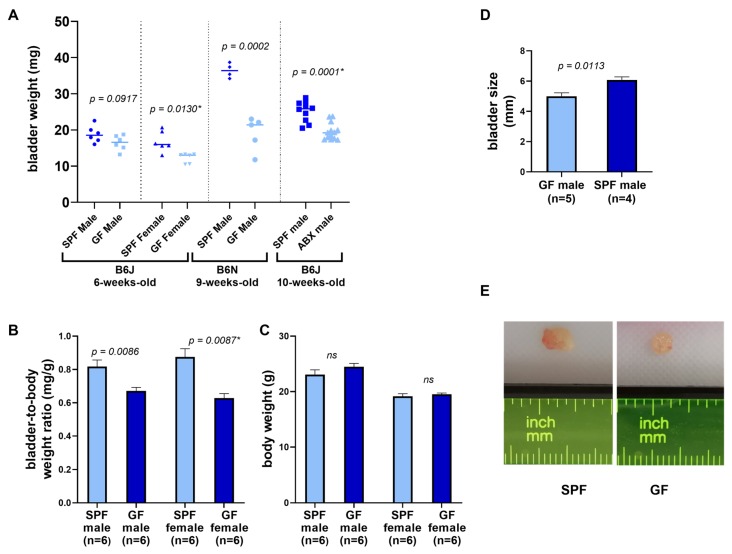
Comparison of bladder weight and size in germ-free and SPF animals. (**A**) Bladder weight of all groups of mice. (**B**) Bladder-to-body weight ratio of B6J 6-weeks-old mice shown in mg/g units. (**C**) Body weight of B6J 6-week-old mice. (**D**) Bladder size (length in mm) of B6N 9-week-old mice. Bars represent mean ± SEM. Student T-test (*p*-value without asterisk) and Mann-Whitney test were used to determine significance (*p*-value with asterisk). (**E**) Representative photographs of GF and SPF bladders. (ABX—antibiotics, ns—non-significant).

A comparison of bladder weight and size between GF and SPF mice is shown in Table 2 and Figure 1. On average, bladders of GF and antibiotic-treated mice weighed 25% less than their age-controlled SPF counterparts. The same was observed with the bladders of antibiotics-treated mice (Table 2, Figure 1A). This difference was significant in case of female B6J mice (*p* = 0.005), male B6N mice (*p* = 0.00003) and male B6J mice treated with antibiotics (*p* = 0.0002). The difference in bladder weight between GF and SPF male mice of B6J substrain was not significant (*p* = 0.09). The biggest difference was observed in B6N group, with GF bladders weighing almost 50% less than the SPF bladders. Germ-free mice can differ from their SPF counterparts in overall body weight, therefore we checked bladder-to-body weight ratio in B6J mice, which was significantly reduced by approximately 20% in male and 30% in female GF animals (Table 2, Figure 1B,C). Germ-free bladders were also smaller in size (Figure 1D,E, and Supplementary Figure S1).

**Figure 2:**
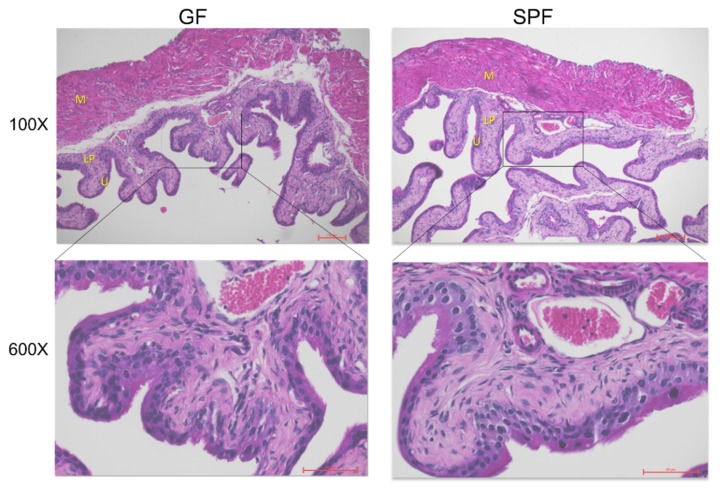
Bladder tissue of GF and SPF animals. Hematoxylin-eosin staining of GF and SPF mice at 100× and 600× magnification. Letters represent particular bladder layers: U—urothelium, LP—lamina propria, M—muscle layer.

Both GF and SPF bladder tissue showed regular bladder tissue composition (Figure 2). The difference in weight was not correlated to any findings in the histological assessment of hematoxylin-eosin stained sections.

### 3.2. Gene Expression

**Figure 3:**
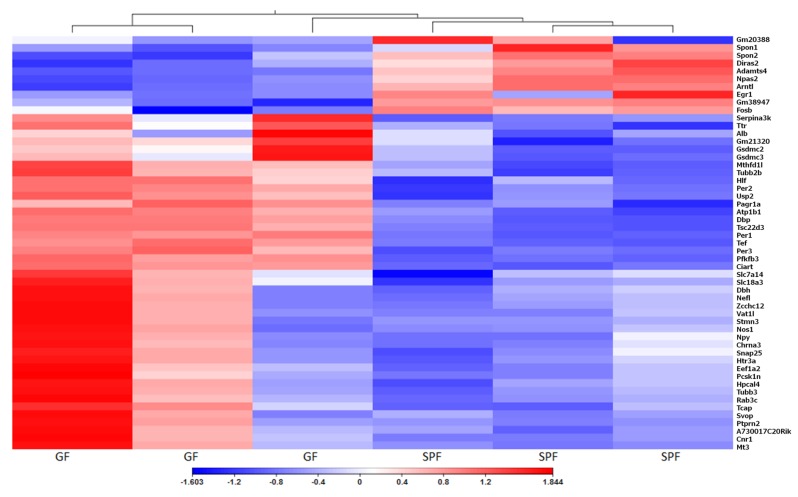
Heat Map of differentially expressed genes. Comparison of urinary bladder gene expression between GF and SPF mice. Values represent log_2_(fold change) with FDR less than 0.01, Euclidian distance, complete linkage.

The transcriptome of the urinary bladder is affected by housing mice under GF conditions. However, principal component analysis (PCA) of GF and SPF bladders did not reveal clear grouping of samples of either group (Supplementary Figure S1). For the differential expression of genes, we used the cut-off of more than a ±2-fold change between groups and FDR *p*-value <0.01. In total, there were 97 differentially expressed genes between GF and SPF groups with 84 genes upregulated and 13 genes downregulated in GF mice versus SPF (Figure 3, Supplementary Table S2). Most down-regulated genes were Gm20388, a gene of unknown function with 44-fold downregulation, Gm38947, also a gene of unknown function with 12-fold downregulation, and Arntl, Aryl hydrocarbon receptor nuclear translocator-like protein 1 that was 4-fold downregulated. Most up-regulated genes were immunoglobulin chain genes Igkv4-68 (159- fold up-regulation), Igkv1-122 (240-fold up-regulation) and Major urinary protein 7 or Mup7 that was 208-fold up-regulated. Results of the qPCR analysis for 13 selected genes correlated with the RNA-seq results. The correlation coefficient (R^2^) for log_2_(fold change) obtained by the two methods was 0.90 and *p*-value < 0.0001 (Supplementary Figure S2). 

There were 25 KEGG pathways that had both significantly up and downregulated gene nodes (Supplementary Table S3). Twenty-five pathways had significantly downregulated gene sets, and 5 pathways had significantly upregulated gene sets (Supplementary Table S3). Gene ontology analysis of differentially expressed genes uncovered enrichment in 14 biological processes including circadian rhythm, synaptic transmission and cell signalling (Table 3). Further, 14 molecular functions were enriched, including acetylcholine receptor activity and neuropeptide hormone activity (Supplementary Table S5). Most enriched cellular components were neuron projection, cell projection and synapse (Supplementary Table S5).

### 3.3. Circadian Rhythm

Several core circadian genes were differentially expressed (mmu04710, Supplementary Figure S3A) between GF and SPF mice. Period genes that play a central role in the molecular circadian clock were upregulated in a bladder of GF mice [15]. Arntl, part of the circadian core clock was downregulated, while Ciart, a circadian associated repressor of transcription, was upregulated in GF mice. Egr1, Fosb, Npas2, and Nfil3 are genes that code for circadian transcription factors and their expression was significantly downregulated in the bladder tissues of germ-free mice. 

### 3.4. Extracellular Matrix

**Figure 4:**
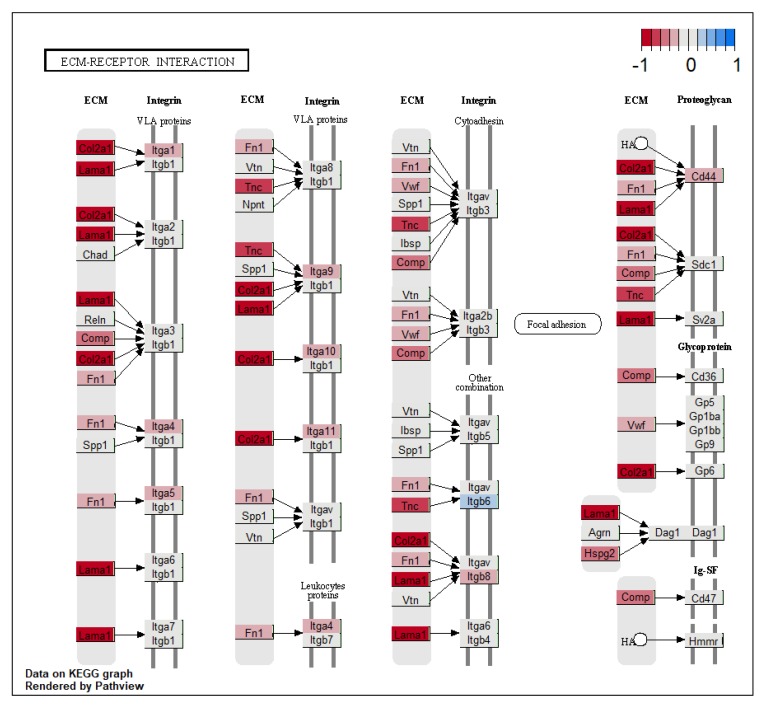
KEGG pathway ‘ECM-receptor interaction’ with significant changes in gene expression.

Several extracellular matrix (ECM) genes were differentially expressed. In the KEGG pathway, ECM-receptor interaction (mmu04512) gene nodes representing accumulative fold change expression of multiple collagen, laminin, and tenascin genes, all coding for proteins of the extracellular matrix that interact with cell surface VLA integrin proteins, were downregulated (Figure 4). Individual gene expression for these genes was not significantly changed in GF tissue. Gene encoding for ECM protein Spondin2 (Spon2) and Spon1 was downregulated in the bladders of germ-free mice compared to SPF. Spon2 is involved in the recognition of bacteria in ECM and the activation of an immune response, while Spon1 has a role in neurogenesis and the maintenance of circadian rhythm [16,17]. Adamts4, a gene with circadian expression, coding for an enzyme involved in cutting aggrecan and cartilage, was downregulated in GF mice. Matrix metalloproteinase 12 (Mmp12), involved in cutting elastin and EMC remodelling was upregulated in GF bladders (Table S2). 

### 3.5. Ion Homeostasis Regulation and Signalling

Genes involved in the regulation of calcium concentrations and calcium signalling in the cell were differentially expressed (mmu04020, Supplementary Figure S3B). Calcium serves as a second messenger that propagates numerous intracellular and extracellular signals, regulating cell proliferation and apoptosis as well as enzyme activity [18]. Stc1 gene has a role in calcium and phosphate cell homeostasis [19], its expression was upregulated in germ-free mice. Calcium channel regulator Rrad gene was downregulated in GF mice, while Acng5, a gene coding for a calcium channel protein, was upregulated in GF mouse bladder tissue. All differentially expressed genes with a calcium binding function are listed in Table 4.

Atp1b1, a gene that encodes for a membrane protein that maintains the gradient of sodium and potassium ions across the cell membrane [20], was upregulated in germ-free mice. Unc80 gene, encoding for part of a complex sodium NALCN channel that transports sodium ions into cells and plays a key role in neuronal cell’s ability to transmit membrane electric signal, was 14-fold upregulated in bladders of GF mice [21]. 

### 3.6. Neuronal Signalling

Six genes expressed in nicotinic acetylcholine neurons were upregulated, including two genes encoding for subunits of cholinergic nicotinic receptors—Neuronal acetylcholine receptor subunit alpha-3 (Chrna3) and subunit beta (Chrnb4), and genes that regulate acetylcholine synthesis and transport into vesicles, namely Solute carrier family 5 member 7 (Slca5a7), Solute carrier family 18 member 3 (Slc18a3), Synaptosome associated protein 25 (Snap25), and Zinc finger CCHC-type containing 12 (Zcchc12) [22,23]. Cannabinoid Receptor 1 (Cnr1), as well as Tyrosine hydroxylase, an important enzyme in the physiology of adrenergic neurons that converts tyrosine to dopamine, had upregulated gene expression in the bladders of germ-free mice [24]. Gene expression of Dbh, Dopamine beta (β)-hydroxylase, an enzyme that converts dopamine to norepinephrine, as well as Svop, synaptic vesicle 2-related protein, 5-hydroxytryptamine receptor 3A and 3B (Htr3a and Htr3b), genes that encode for heterodimer receptor for serotonin, were all upregulated in GF mice [25]. Genes for Protein tyrosine phosphatase receptor type N and type N2 (Ptprn and Ptprn2) that regulate secretion from vesicles and might be involved in the regulation of secretion of neurotransmitters, were upregulated in GF bladders [26]. Neurofilament Light gene (Nefl), involved in neurofilament formation, was upregulated in GF mice as well. Gene for vasoactive intestinal peptide (Vip), a neuropeptide known to control circadian expression in the suprachiasmatic nucleus, the part of the brain that coordinates rhythmic behaviour, was nine-fold upregulated in GF mice [27]. All neuronal associated and neuronal specific genes differentially expressed in germ-free bladders are listed in Table 5. 

### 3.7. Regulation of Detoxification of Xenobiotics

PAR bZip transcription factors, Tef, Hlf and Dbp that regulate the expression of phase 1, 2 and 3 detoxifying enzymes and are the primary sensors of xenobiotics [28], had higher gene expression in the tissues of GF mice. 

### 3.8. Immune System

Since GF animals are devoid of bacteria, we expected to see differences in pathogen recognition receptors and their signalling molecules. However, none of the innate immunity pattern recognition receptors were differentially expressed (Table S7). B receptor signalling pathway (mmu04662) was perturbed with several gene nodes involved in calcium signalling and MAPK pathway differentially expressed in germ-free bladders (Figure S3C). Three genes coding for proteins with anti-inflammatory or immunosuppressive function were upregulated in GF bladders: TSC22 Domain Family Member 3 (Tsc22d3), Alpha-1-acid glycoprotein 1 (Orm1) and Serine (or cysteine) peptidase inhibitor, clade A, member 3K (Serpina3k). Six immunoglobulin chain genes were upregulated in the germ-free bladders, including light kappa and lambda chains as well as several heavy chains, some over 200-fold change (Table 6) which represents the highest change in gene expression in this study. 

### 3.9. Other Genes

Gasdermin genes Gsdmc3 and Gsdmc2 have the highest expression in bladder tissue. Their protein products promote pyroptosis and exhibit bactericidal activity [29]. Germ-free conditions lead to upregulation of these proteins in the bladder. Gene for Mup7, one of the major urinary proteins, the most abundant proteins in the mouse urine, was 200-fold upregulated in germ-free conditions, as well as another major urinary protein, Mup22 or Gm21320 (23-fold). 

## 4. Discussion

Because the evidence for the existence of the microbiome in the urinary bladder is relatively new, its role in the organ’s health and disease is poorly understood. An increasing number of studies show a link between the urinary microbiome and bladder health, the best characterized connection is between bladder dysbiosis and its cancer [30,31]. To our knowledge, this is the first study showing that the microbiota influences gene expression in the urinary bladder and, surprisingly, it also controls urinary bladder weight and size. Some of the gene expression changes, e.g., change of circadian rhythm genes, were previously noted in the gut, but some changes, like the difference in extracellular matrix, immune, or neuronal genes are more novel.

The GF urinary bladders had on average 25% lower weight than bladders from mice housed under standard SPF conditions. This result was obtained from GF and SPF mice from two different facilities and of two different C57BL/6 substrains. Although GF animals are known to have reduced fat tissue deposition or an enlarged cecum, this is the first report of reduced bladder weight and size [6]. Careful pathohistological analysis of tissue specimens did not reveal any structural differences in bladder tissue between bladders of GF and SPF mice. In order to get insight into possible reasons for reduced bladder weight and size among GF mice, we analysed the expression of genes known to play a role in bladder development. We presumed that the absence of microbiota during embryonic growth might result in reduced bladder weight and size. Thus, we checked the expression of genes involved in urinary bladder development in the RNA-seq dataset [32]. Among all genes involved in embryonic development of urinary bladder, only the gene coding for Veriscan, an ECM proteoglycan, was significantly downregulated (Supplementary Figure S4C). Since Versican is only associated with a particular eye disorder, vitreoretinopathy, it is most probably not responsible for the observed reduced bladder size and weight [33]. To confirm that reduced bladder weight is not a microbiota-driven developmental anomaly, we checked whether it will be possible to induce such a change in mice exposed to standard microbiota. To that aim, we depleted microbiota among SPF housed mice by the use of broad-spectrum antibiotics. Surprisingly, we reproduced reduced bladder weight phenotype also in SPF mice. This indicates that a reduction in bladder weight is not developmentally determined, but is rather an adaptive bladder change in response to the absence of microbiota or their products. To assess differences in the composition of cells that make up bladder tissue, we compared the expression of gene markers for bladder cell types, but found no significant changes (Supplementary Figure S4A) [34]. It has been reported that microbiota controls muscle size and function with GF mice having smaller muscle mass and showing more muscle atrophy [35]. Since muscle constitutes most of the bladder mass, we checked the expression of genes controlling muscle size [36], in order to see if its decrease is involved in reduced bladder weight. However, we did not find any differentially expressed genes (Supplementary Figure S4B), leaving open the question of transcriptional change that might be driving observed differences. Another explanation for the weight difference could be the difference in the composition of the extracellular matrix (ECM), another tissue component that constitutes a significant proportion of bladder mass. Cumulative expression of a group of genes involved in ECM physiology (collagens, tenascins and laminins) was downregulated in GF mice and ECM degrading enzyme Mmp12 was upregulated. These changes in the protein composition of the extracellular matrix could disturb the water and polysaccharide content of ECM and possibly change the overall weight of the tissue. Finally, it is possible that the difference in weight might be also due to changes at post-transcriptional level which we cannot explain with only RNA-seq data [37]. Bearing in mind that overall bladder histology was not altered in GF mice, it is reasonable to hypothesize that different bladder components were proportionally reduced by a cumulative effect of complex regulatory network, controlled by microbiota, in which ECM genes may play a leading role. 

Circadian rhythm gene expression was changed in the bladders of GF mice, which is in concordance with several studies showing that gut microbiota regulates circadian programming of the host [38,39]. The timing of food consumption, as well as diet, control the oscillations in microbiome that, in turn, control circadian rhythm changes [40]. These circadian oscillations are heavily perturbed in mice lacking microbiome or in mice with antibiotics-reduced microbiome [40]. Circadian period genes were significantly upregulated in the bladders of GF mice in this study, while Nfil3, a negative regulator of transcription of Per1 and Per2, was downregulated in GF bladders [39]. Mice knockout for Per1 and Per2 have an arrhythmic bladder voiding schedule, demonstrating that those circadian oscillators control the pattern of bladder voiding [41]. Our results show that the microbiota affects the circadian gene expression in the urinary bladder, suggesting that microbiota has a role in diurnal bladder voiding. 

Several genes involved in neuronal functioning were upregulated in GF mouse bladders. It is known that gut microbiota can affect the development and function of the nervous system through the release of various metabolites and modulation of immune cells [42]. The gut–brain axis is the two-way communication between gut bacteria and the brain, with the microbiota influencing brain function and behaviour of individuals [42]. In the urinary bladder, sympathetic and parasympathetic nerves innervating bladder muscles control urine storage and elimination, functions mediated by cholinergic nicotinic receptors [43]. Genes encoding for subunits of those receptors, as well for enzymes involved in acetylcholine synthesis and secretion were upregulated in GF bladders. Furthermore, cholinergic enzyme activity, receptor expression, and acetylcholine release fluctuate in a circadian manner and are known to be under the control of circadian oscillators [44]. These changes, together with observed changes in circadian rhythm genes may explain circadian micturition pattern and might indicate the existence of bladder-brain axis. 

The link between microbiota and the expression of major urinary proteins (Mups), the most abundant proteins in mouse urine, has been described previously [45]. Mups function as transporters of pheromones that are responsible for urinary odour can trigger male aggression [45]. They are mainly produced in the liver, where they are downregulated in mice lacking microbiota, which leads to a lower abundance of Mups in the urine of GF mice [46]. In this study, we have found that the Mup7 and Mup22 (predicted gene Gm21320) genes were overexpressed in the bladders of GF mice, which could be a compensation mechanism for their reduced expression in the liver.

Another interesting finding was the significant upregulation of expression of six immunoglobulin chain genes in germ-free bladders. This could be due to the fact that germ-free mice have increased bone mass and bone marrow, the reservoir for immunoglobulin producing plasma cells [47]. Several gene markers for B cells were upregulated in GF bladders, but not significantly (Supplementary Figure S4A). Besides bone marrow plasma cells, some epithelial cells and tumour cells can also produce Ig chain genes [48,49]. It is not known if healthy urothelial cells can produce immunoglobulins and what would be their function in the bladder and whether they are modulated by microbiota. 

Both mouse bladder function and transcriptome change [50], with the onset of the impaired contractile property of the bladder and lower gene expression of muscarinic, purinergic, and β-adrenergic receptors in aged mice compared to mature mice. Bladder weight and bladder-to-body weight ratio increases with age. Our study revealed the upregulation of cholinergic receptors as well as lower bladder weight and bladder-to-body weight ratio in germ-free mice, which would suggest GF bladders resembling the bladder profile of younger mice in comparison to the SPF bladders.

This study provides additional evidence that microbiota has a profound effect on the organisms it inhabits. Since germ-free mice are devoid of gut and bladder microbiota, it is impossible to distinguish the effect of urinary microbiota from the gut microbiota in these results. However, we demonstrated that removing all microbiota from the body modifies gene expression in the urinary bladder and, surprisingly, affects urinary bladder weight and size. Although we have shown some very interesting findings, this study is mainly descriptive in its nature. To elucidate further mechanisms and the physiological importance of observed transcriptional and organ size changes, warrants additional, mechanistically-oriented studies. 

## Figures and Tables

**Table 1:** List of all primer sequences used for qPCR analysis.

Primer	DNA Sequence (5’–3’)
mSpon2 F	TTGCCAGGTGATGGAAAACG
mSpon2 R	CGGGCTGTACAAACCGATTC
mAdamts4 F	TGTCATGGCTCCTGTCATGG
mAdamts4 R	AGGCAGTGCCCATAACCATT
mPer1 F	CTCCTGCTCCAGTGACTTTCC
mPer1 R	GGCTTGGCCCGAGATTCAA
mArntl F	GTAGATCAGAGGGCGACAGC
mArntl R	CCTGTGACATTCTGCGAGGT
mTef F	TGTCCAGCACAGAATCGTCC
mTef R	GCAGGGTCAGGGTTGAAGTT
mPer2 F	CCATCCACAAGAAGATCCTAC
mPer2 R	GCTCCACGGGTTGATGAAGC
mRev-erba F	ACATGTATCCCCATGGACGC
mRev-erba R	CTGGTCGTGCTGAGAAAGGT
mNfil3 F	CTTTCAGGACTACCAGACATCCAA
mNfil3 R	GATGCAACTTCCGGCTACCA
mPer3 F	GAGAGGCACACTAAGCCCAG
mPer3 R	GCCGCGAAGGTATCTGTGTT
mCol2a1 F	GAGGCGATGTTGGCGAGAAA
mCol2a1 R	GAGGTCCGACTTCTCCCTTC
mLama1 F	GGTCATGCAGAGGCTGACTT
mLama1 R	TGCTGTCAGCTTGTTTCCGA
mTnc F	AACGGACTGCCCACATCTCA
mTnc R	TCCGGTTCAGCTTCTGTGGTAG
mEgfr F	TCATCTGTGCCCAGCAATGT
mEgfr R	TTGGCAGACCAGACAGTCAC
mCldn1 F	TGGGGCTGATCGCAATCTTT
mCldn1 R	CACTAATGTCGCCAGACCTGA
mActb F	CACTGTCGAGTCGCGTCC
mAtcb R	TCATCCATGGCGAACTGGTG

**Table 2:** General characteristics of analyzed mice.

Microbiota Status	Facility	Strain	No. Animals in Group	Age (Weeks)	Sex	bladder Weight (mg) ^d^	*p* Value ^e^	Bladder-to-Body Weight Ratio ^d^	*p* Value ^e^
SPF	IGCa	B6J	6	6	male	18.82 ± 2.31	0.09175	1.24 ± 0.14	0.0086
GF	IGC	B6J	6	6	male	16.45 ± 2.09		1.5 ± 0.12	
SPF	IGC	B6J	6	6	female	16.82 ± 2.83	0.0130	1.16 ± 0.17	0.0087
GF	IGC	B6J	6	6	female	12.28 ± 1.35		1.61 ± 0.18	
SPF	HZIb	B6N	4	9	male	36.43 ± 2.01	0.00023	-	-
GF	HZI	B6N	5	9	male	19.1 ± 4.65			
Control (SPF)	USSMc	B6J	10	11	male	25.17 ± 2.85	0.0001		
Antibiotics reduction	USSM	B6J	13	11	male	19.5 ± 2.33		-	-

^a^ Instituto Gulbenkian de Ciência, ^b^ Helmholtz Centre for Infection, ^c^ University of Split School of Medicine, ^d^ mean ± SD, ^e^ *p*-value was calculated using Student T-Test for the first and third group and Mann-Whitney rank test for the second and fourth group of mice.

**Table 3:** List of biological processes enriched in GF bladder tissue obtained by Gene Ontology analysis.

GO Biological Process	# (Total Number in Reference Genome)	# (Total Number of Genes in Our Dataset)	Fold Enrichment	*p*-Value	FDR *p*-Value
circadian rhythm	13	4	71.35	7.28 × 10^−7^	5.92 × 10^−5^
rhythmic process	13	4	71.35	7.28 × 10^−7^	4.44 × 10^−5^
neuromuscular synaptic transmission	47	4	19.74	6.83 × 10^−5^	2.78 × 10^−3^
synaptic transmission	392	15	8.87	2.11 × 10^−10^	2.58 × 10^−8^
cell-cell signaling	588	18	7.1	9.30 × 10^−11^	2.27 × 10^−8^
cell communication	3269	28	1.99	2.48 × 10^−4^	6.06 × 10^−3^
cellular process	8762	58	1.54	3.64 × 10^−5^	1.78 × 10^−3^
synaptic vesicle exocytosis	58	3	11.99	2.31 × 10^−3^	3.75 × 10^−2^
neurological system process	1393	16	2.66	3.13 × 10^−4^	6.94 × 10^−3^
system process	1487	17	2.65	2.09 × 10^−4^	5.67 × 10^−3^
single-multicellular organism process	2258	23	2.36	1.08 × 10^−4^	3.77 × 10^−3^
multicellular organismal process	2274	23	2.35	1.14 × 10^−4^	3.49 × 10^−3^
response to endogenous stimulus	229	6	6.08	5.33 × 10^−4^	1.08 × 10^−2^
biosynthetic process	1719	17	2.29	1.50 × 10^−3^	2.61 × 10^−2^

**Table 4:** Differentially expressed genes involved in the regulation of calcium binding and signalling.

Gene Symbol	Identifier	Gene Name	Mean Total Counts ^a^	Fold Change	FDR *p*-Value
Rrad	ENSMUSG00000031880	Ras-related associated with diabetes	788	−2.1	1.2 × 10^−3^
Stc1	ENSMUSG00000014813	stanniocalcin 1	395	2.0	1.1 × 10^−3^
Syt4	ENSMUSG00000024261	synaptotagmin IV	24	7.9	5.1 × 10^−3^
Mmp12	ENSMUSG00000049723	matrix metallopeptidase 12	35	8.0	2.5 × 10^−3^
Alb	ENSMUSG00000029368	albumin	16	12.4	8.4 × 10^−4^
Hpcal4	ENSMUSG00000046093	hippocalcin-like 4	10	13.2	3.6 × 10^−4^
Fstl5	ENSMUSG00000034098	follistatin-like 5	5	13.8	2.5 × 10^−3^
Cacng5	ENSMUSG00000040373	calcium channel, voltage-dependent, gamma subunit 5	6	16.3	6.6 × 10^−3^

^a^ Mean Total Counts is the mean of a normalized number of reads for each gene in the three SPF samples.

**Table 5:** Differentially expressed genes specific for or associated with neural tissue.

Gene Symbol	Identifier	Gene Name	Mean Total Counts ^a^	Fold Change	FDR *p*-Value
Tubb2b	ENSMUSG00000045136	tubulin, beta 2B class IIB	179	2.9	1.2 × 10^−6^
Mapk10	ENSMUSG00000046709	mitogen-activated protein kinase 10	22	4.7	9.0 × 10^−3^
Elavl2	ENSMUSG00000008489	ELAV (embryonic lethal, abnormal vision, Drosophila)-like 2 (Hu antigen B)	38	5.0	5.3 × 10^−3^
Ptprn	ENSMUSG00000026204	protein tyrosine phosphatase, receptor type, N	45	5.5	8.7 × 10^−3^
Ngfr	ENSMUSG00000000120	nerve growth factor receptor (TNFR superfamily, member 16)	220	5.7	1.1 × 10^−3^
Prph	ENSMUSG00000023484	peripherin	156	5.7	1.3 × 10^−3^
Gap43	ENSMUSG00000047261	growth associated protein 43	61	5.9	1.8 × 10^−3^
Chgb	ENSMUSG00000027350	chromogranin B	10	6.0	7.2 × 10^−3^
Cnr1	ENSMUSG00000044288	cannabinoid receptor 1 (brain)	18	7.2	8.3 × 10^−4^
Nos1	ENSMUSG00000029361	nitric oxide synthase 1, neuronal	55	7.7	7.1 × 10^−4^
Syt4	ENSMUSG00000024261	synaptotagmin IV	24	7.9	5.1 × 10^−3^
Ptprn2	ENSMUSG00000056553	protein tyrosine phosphatase, receptor type, N polypeptide 2	17	8.0	7.7 × 10^−4^
Slc5a7	ENSMUSG00000023945	solute carrier family 5 (choline transporter), member 7	19	8.5	1.8 × 10^−3^
Nefl	ENSMUSG00000022055	neurofilament, light polypeptide	53	8.9	2.1 × 10^−4^
Snap25	ENSMUSG00000027273	synaptosomal-associated protein 25	57	8.9	9.2 × 10^−4^
Vip	ENSMUSG00000019772	vasoactive intestinal polypeptide	45	9.4	1.8 × 10^−3^
Slc18a3	ENSMUSG00000100241	solute carrier family 18 (vesicular monoamine), member 3	9	9.4	7.1 × 10^−4^
Cartpt	ENSMUSG00000021647	CART prepropeptide	19	9.7	2.3 × 10^−3^
Vstm2l	ENSMUSG00000037843	V-set and transmembrane domain containing 2-like	14	10.2	8.7 × 10^−3^
Cplx1	ENSMUSG00000033615	complexin 1	14	10.2	5.1 × 10^−3^
Htr3a	ENSMUSG00000032269	5-hydroxytryptamine (serotonin) receptor 3A	57	10.2	1.1 × 10^−4^
Kcnq2	ENSMUSG00000016346	potassium voltage-gated channel, subfamily Q, member 2	10	10.4	2.8 × 10^−3^
Htr3b	ENSMUSG00000008590	5-hydroxytryptamine (serotonin) receptor 3B	8	10.7	1.5 × 10^−3^
Vat1l	ENSMUSG00000046844	vesicle amine transport protein 1 like	75	11.2	2.2 × 10^−5^
Npy	ENSMUSG00000029819	neuropeptide Y	27	11.8	7.4 × 10^−4^
Zcchc12	ENSMUSG00000036699	zinc finger, CCHC domain containing 12	13	11.9	7.3 × 10^−4^
Jph3	ENSMUSG00000025318	junctophilin 3	5	12.3	1.3 × 10^−3^
Chrna3	ENSMUSG00000032303	cholinergic receptor, nicotinic, alpha polypeptide 3	19	12.6	7.5 × 10^−4^
Chrnb4	ENSMUSG00000035200	cholinergic receptor, nicotinic, beta polypeptide 4	7	13.1	3.3 × 10^−3^
Th	ENSMUSG00000000214	tyrosine hydroxylase	10	13.4	4.4 × 10^−3^
Dbh	ENSMUSG00000000889	dopamine beta hydroxylase	20	14.2	2.2 × 10^−4^
Ctnna2	ENSMUSG00000063063	catenin (cadherin associated protein), alpha 2	4	14.3	1.1 × 10^−3^
Tlx2	ENSMUSG00000068327	T cell leukemia, homeobox 2	4	15.2	6.2 × 10^−3^
Gria2	ENSMUSG00000033981	glutamate receptor, ionotropic, AMPA2 (alpha 2)	2	25.5	1.4 × 10^−3^
Svop	ENSMUSG00000042078	SV2 related protein	2	32.8	6.9 × 10^−4^

^a^ Mean Total Counts is the mean of a normalized number of reads for each gene in the three SPF samples.

**Table 6:** List of differentially expressed immunoglobulin chain genes.

Gene Symbol	Identifier	Gene Name	Mean Total Counts ^a^	Fold Change	FDR *p*-Value
Iglc2	ENSMUSG00000076937	immunoglobulin lambda constant 2	4	14.4	6.0 × 10^−3^
Igkv15-103	ENSMUSG00000076523	immunoglobulin kappa chain variable 15-103	7	20.7	2.4 × 10^−3^
Iglv2	ENSMUSG00000076940	immunoglobulin lambda variable 2	1	60.2	1.9 × 10^−3^
Ighv1-36	ENSMUSG00000094051	immunoglobulin heavy variable 1-36	0.3	159.0	6.3 × 10^−3^
Igkv4-68	ENSMUSG00000076549	immunoglobulin kappa variable 4-68	0.3	159.5	3.8 × 10^−3^
Igkv1-122	ENSMUSG00000095497	immunoglobulin kappa chain variable 1-122	0.3	240.3	1.2 × 10^−3^

^a^ Mean Total Counts is the mean of a normalized number of reads for each gene in the three SPF samples.

## Supplementary Materials

The following are available online at https://www.mdpi.com/2076-2607/8/3/421/s1, Figure S1: RNA-Seq sample comparison of GF and SPF groups, Figure S2: Concurrence of log_2_(fold change) from qPCR and RNA-seq analysis for 13 exemplar genes, Figure S3: KEGG pathways with significant change in gene expression, Figure S4: Log_2_(fold change (GF vs SPF)) of gene expression for A. gene markers for bladder cell types B. genes controlling muscle growth C. genes involved in bladder development, Table S1: Summary of mapping reads to mouse genome, Table S2: All differentially expressed genes, Table S3: All KEGG pathways with significant changes in expression, Table S4: Enriched Molecular Functions in GF bladders, Table S5: Cellular components, reactome pathways and protein class enriched in GF tissue, Table S6: Shapiro-Wilk test for normal distribution, Table S7: Expression of genes for pattern recognition receptors.

## References

[1] Cho I., Blaser M.J. (2012). The human microbiome: At the interface of health and disease. Nat. Rev. Genet..

[2] Wojciuk B., Salabura A., Grygorcewicz B., Kędzierska K., Ciechanowski K., Dołęgowska B. (2019). Urobiome: In Sickness and in Health. Microorganisms.

[3] Redelman-Sidi G., Glickman M.S., Bochner B.H. (2014). The mechanism of action of BCG therapy for bladder cancer-A current perspective. Nat. Rev. Urol..

[4] Al-Asmakh M., Zadjali F. (2015). Use of germ-free animal models in microbiota-related research. J. Microbiol. Biotechnol..

[5] Bleich A., Fox J.G. (2015). The mammalian microbiome and its importance in laboratory animal research. ILAR J..

[6] Smith K., McCoy K.D., Macpherson A.J. (2007). Use of axenic animals in studying the adaptation of mammals to their commensal intestinal microbiota. Semin. Immunol..

[7] Grivennikov S.I., Wang K., Mucida D., Stewart C.A., Schnabl B., Jauch D., Taniguchi K., Yu G.Y., Österreicher C.H., Hung K.E. (2012). Adenoma-linked barrier defects and microbial products drive IL-23/IL-17-mediated tumour growth. Nature.

[8] Trapnell C., Williams B.A., Pertea G., Mortazavi A., Kwan G., Van Baren M.J., Salzberg S.L., Wold B.J., Pachter L. (2010). Transcript assembly and quantification by RNA-Seq reveals unannotated transcripts and isoform switching during cell differentiation. Nat. Biotechnol..

[9] Love M.I., Huber W., Anders S. (2014). Moderated estimation of fold change and dispersion for RNA-seq data with DESeq2. Genome Biol..

[10] Kanehisa M. (2000). KEGG: Kyoto Encyclopedia of Genes and Genomes. Nucleic Acids Res..

[11] R Core Team (2019). R: A Language and Environment for Statistical Computing, Version 3.5.0.

[12] Luo W., Friedman M.S., Shedden K., Hankenson K.D., Woolf P.J. (2009). GAGE: Generally applicable gene set enrichment for pathway analysis. BMC Bioinform..

[13] Luo W., Brouwer C. (2013). Pathview: An R/Bioconductor package for pathway-based data integration and visualization. Bioinformatics.

[14] Hrdý J., Novotná O., Kocourková I., Prokešová L. (2012). Cytokine expression in the colostral cells of healthy and allergic mothers. Folia Microbiol..

[15] Takahashi J.S. (2017). Transcriptional architecture of the mammalian circadian clock. Nat. Rev. Genet..

[16] He Y.-W., Li H., Zhang J., Hsu C.-L., Lin E., Zhang N., Guo J., Forbush K.A., Bevan M.J. (2004). The extracellular matrix protein mindin is a pattern-recognition molecule for microbial pathogens. Nat. Immunol..

[17] Carrillo G.L., Su J., Monavarfeshani A., Fox M.A. (2018). F-spondin is essential for maintaining circadian rhythms. Front. Neural Circuits.

[18] Clapham D.E. (2007). Calcium Signaling. Cell.

[19] Xiang J., Guo R., Wan C., Wu L., Yang S., Guo D. (2016). Regulation of intestinal epithelial calcium transport proteins by stanniocalcin-1 in Caco2 cells. Int. J. Mol. Sci..

[20] Li Y., Yang J., Li S., Zhang J., Zheng J., Hou W., Zhao H., Guo Y., Liu X., Dou K. (2011). N-myc downstream-regulated gene 2, a novel estrogen-targeted gene, is involved in the regulation of Na + /K + -ATPase. J. Biol. Chem..

[21] Lu B., Zhang Q., Wang H., Wang Y., Nakayama M., Ren D. (2010). Extracellular Calcium Controls Background Current and Neuronal Excitability via an UNC79-UNC80-NALCN Cation Channel Complex. Neuron.

[22] Barwick K.E.S., Wright J., Al-Turki S., McEntagart M.M., Nair A., Chioza B., Al-Memar A., Modarres H., Reilly M.M., Dick K.J. (2012). Defective presynaptic choline transport underlies hereditary motor neuropathy. Am. J. Hum. Genet..

[23] O’Grady G.L., Verschuuren C., Yuen M., Webster R., Menezes M., Fock J.M., Pride N., Best H.A., Benavides Damm T., Turner C. (2016). Variants in SLC18A3, vesicular acetylcholine transporter, cause congenital myasthenic syndrome. Neurology.

[24] Tolleson C., Claassen D. (2012). The Function of Tyrosine Hydroxylase in the Normal and Parkinsonian Brain. CNS Neurol. Disord. Drug Targets.

[25] Rush R.A., Geffen L.B. (1980). Dopamine βhydroxylase in health and disease. Crit. Rev. Clin. Lab. Sci..

[26] Mandemakers W., Abuhatzira L., Xu H., Caromile L.A., Hébert S.S., Snellinx A., Morais V.A., Matta S., Cai T., Notkins A.L. (2013). Co-regulation of intragenic microRNA miR-153 and its host gene Ia-2 β: Identification of miR-153 target genes with functions related to IA-2β in pancreas and brain. Diabetologia.

[27] Hamnett R., Crosby P., Chesham J.E., Hastings M.H. (2019). Vasoactive intestinal peptide controls the suprachiasmatic circadian clock network via ERK1/2 and DUSP4 signalling. Nat. Commun..

[28] Michalik L., Auwerx J., Berger J.P., Chatterjee V.K., Glass C.K., Gonzalez F.J., Grimaldi P.A., Kadowaki T., Lazar M.A., O’Rahilly S. (2006). International union of pharmacology. LXI. Peroxisome proliferator-activated receptors. Pharmacol. Rev..

[29] Shi J., Gao W., Shao F. (2017). Pyroptosis: Gasdermin-Mediated Programmed Necrotic Cell Death. Trends Biochem. Sci..

[30] Bajic P., Wolfe A.J., Gupta G.N. (2019). The Urinary Microbiome: Implications in Bladder Cancer Pathogenesis and Therapeutics. Urology.

[31] Bučević Popović V., Šitum M., Chow C.E.T., Chan L.S., Roje B., Terzić J. (2018). The urinary microbiome associated with bladder cancer. Sci. Rep..

[32] Price K.L., Woolf A.S., Long D.A. (2009). Unraveling the Genetic Landscape of Bladder Development in Mice. J. Urol..

[33] Kloeckener-Gruissem B., Neidhardt J., Magyar I., Plauchu H., Zech J.C., Morlé L., Palmer-Smith S.M., MacDonald M.J., Nas V., Fry A.E. (2013). Novel VCAN mutations and evidence for unbalanced alternative splicing in the pathogenesis of Wagner syndrome. Eur. J. Hum. Genet..

[34] Yu Z., Liao J., Chen Y., Zou C., Zhang H., Cheng J., Liu D., Li T., Zhang Q., Li J. (2019). Single-cell transcriptomic map of the human and mouse bladders. J. Am. Soc. Nephrol..

[35] Lahiri S., Kim H., Garcia-Perez I., Reza M.M., Martin K.A., Kundu P., Cox L.M., Selkrig J., Posma J.M., Zhang H. (2019). The gut microbiota influences skeletal muscle mass and function in mice. Sci. Transl. Med..

[36] Verbrugge S.A.J., Schönfelder M., Becker L., Nezhad F.Y., de Angelis M.H., Wackerhage H. (2018). Genes whose gain or loss-of-function increases skeletal muscle mass in mice: A systematic literature review. Front. Physiol..

[37] Qiao L.Y., Xia C., Shen S., Lee S.H., Ratz P.H., Fraser M.O., Miner A., Speich J.E., Lysiak J.J., Steers W.D. (2018). Urinary bladder organ hypertrophy is partially regulated by Akt1-mediated protein synthesis pathway. Life Sci..

[38] Parkar S., Kalsbeek A., Cheeseman J. (2019). Potential Role for the Gut Microbiota in Modulating Host Circadian Rhythms and Metabolic Health. Microorganisms.

[39] Wang Y., Kuang Z., Yu X., Ruhn K.A., Kubo M., Hooper L.V. (2017). The intestinal microbiota regulates body composition through NFIL3 and the circadian clock. Science.

[40] Thaiss C.A., Levy M., Korem T., Dohnalová L., Shapiro H., Jaitin D.A., David E., Winter D.R., Gury-BenAri M., Tatirovsky E. (2016). Microbiota Diurnal Rhythmicity Programs Host Transcriptome Oscillations. Cell.

[41] Noh J.Y., Han D.H., Kim M.H., Ko I.G., Kim S.E., Park N., Choe H.K., Kim K.H., Kim K., Kim C.J. (2014). Presence of multiple peripheral circadian oscillators in the tissues controlling voiding function in mice. Exp. Mol. Med..

[42] Sharon G., Sampson T.R., Geschwind D.H., Mazmanian S.K. (2016). The Central Nervous System and the Gut Microbiome. Cell.

[43] Sellers D.J., Chess-Williams R. (2012). Muscarinic agonists and antagonists: Effects on the urinary bladder. Handb. Exp. Pharmacol..

[44] Robertson A.G., Kim J., Al-Ahmadie H., Bellmunt J., Guo G., Cherniack A.D., Hinoue T., Laird P.W., Hoadley K.A., Akbani R. (2017). Comprehensive Molecular Characterization of Muscle-Invasive Bladder Cancer. Cell.

[45] Chamero P., Marton T.F., Logan D.W., Flanagan K., Cruz J.R., Saghatelian A., Cravatt B.F., Stowers L. (2007). Identification of protein pheromones that promote aggressive behaviour. Nature.

[46] Weger B.D., Dric Gobet C., Yeung J., Chou J., Naef F. (2019). The Mouse Microbiome Is Required for Sex-Specific Diurnal Rhythms of Gene Expression and Metabolism. Cell Metab..

[47] Sjögren K., Engdahl C., Henning P., Lerner U.H., Tremaroli V., Lagerquist M.K., Bäckhed F., Ohlsson C. (2012). The gut microbiota regulates bone mass in mice. J. Bone Miner. Res..

[48] Hu F., Zhang L., Zheng J., Zhao L., Huang J., Shao W., Liao Q., Ma T., Geng L., Yin C.C. (2012). Spontaneous Production of Immunoglobulin M in Human Epithelial Cancer Cells. PLoS ONE.

[49] Sheng Z., Liu Y., Qin C., Liu Z., Yuan Y., Yin H., Qiu X., Xu T. (2016). Involvement of cancer-derived IgG in the proliferation, migration and invasion of bladder cancer cells. Oncol. Lett..

[50] Kamei J., Ito H., Aizawa N., Hotta H., Kojima T., Fujita Y., Ito M., Homma Y., Igawa Y. (2018). Age-related changes in function and gene expression of the male and female mouse bladder. Sci. Rep..

