# Supplementary material for: Microbiota Alters Urinary Bladder Weight and Gene Expression
Source: Microorganisms. 2020 Mar 17;8(3):421. doi: 10.3390/microorganisms8030421 (PMC7143536; doi:10.3390/microorganisms8030421)
Supplement: Supplementary file 1 [file microorganisms-08-00421-s001.pdf]

microorganisms

MDPI

**Table S1.** Summary of mapping reads to mouse genome.

| Sample Name | Total Reads | Total Mapped | Multiple Mapped | Uniquely Mapped |
|---|---|---|---|---|
| Germ-free Sample 1 | 98926086 | 96.20% | 6.29% | 89.91% |
| Germ-free Sample 2 | 94561652 | 96.58% | 5.83% | 90.75% |
| Germ-free Sample 3 | 88830420 | 96.68% | 6.54% | 90.15% |
| SPF Sample 1 | 103975920 | 96.11% | 5.41% | 90.70% |
| SPF sample 2 | 94930984 | 95.80% | 5.47% | 90.33% |
| SPF Sample 3 | 79055336 | 96.14% | 7.26% | 88.87% |

**Table S2.** All differentially expressed genes.

| *Gene symbol* | | *Gene name* | *GF vs. SPF Fold change* | *FDR p-value* |
|---|---|---|---|---|
| *Gm20388* | ENSMUSG00000092329 | predicted gene | -44.76 | 4.50E-05 |
| *Gm38947* | ENSMUSG00000110365 | predicted gene | -12.14 | 7.60E-06 |
| *Arntl* | ENSMUSG00000055116 | aryl hydrocarbon receptor nuclear translocator-like | -4.36 | 0.00E+00 |
| *Adamts4* | ENSMUSG00000006403 | a disintegrin-like and metallopeptidase | -3.87 | 1.80E-09 |
| *Cyp26b1* | ENSMUSG00000063415 | cytochrome P450, family 26, subfamily b, polypeptide 1 | -3.5 | 2.00E-03 |
| *Diras2* | ENSMUSG00000047842 | DIRAS family, GTP-binding RAS-like 2 | -3.14 | 3.90E-08 |
| *Egr1* | ENSMUSG00000038418 | early growth response 1 | -3.06 | 2.10E-04 |
| *Fosb* | ENSMUSG00000003545 | FBJ osteosarcoma oncogene B | -2.81 | 8.30E-04 |
| *Spon2* | ENSMUSG00000037379 | spondin 2, extracellular matrix protein | -2.73 | 7.20E-13 |
| *Npas2* | ENSMUSG00000026077 | neuronal PAS domain protein 2 | -2.61 | 8.10E-09 |
| *Nfil3* | ENSMUSG00000056749 | nuclear factor, interleukin 3, regulated | -2.2 | 3.00E-03 |
| *Rrad* | ENSMUSG00000031880 | Ras-related associated with diabetes | -2.1 | 1.20E-03 |
| *Spon1* | ENSMUSG00000038156 | spondin 1, (f-spondin) extracellular matrix protein | -2.04 | 5.20E-05 |
| *Stc1* | ENSMUSG00000014813 | stanniocalcin 1 | 2 | 1.10E-03 |
| *Atp1b1* | ENSMUSG00000026576 | ATPase, Na+/K+ transporting, beta 1 polypeptide | 2.01 | 3.20E-09 |
| *Mthfd1l* | ENSMUSG00000040675 | methylenetetrahydrofolate dehydrogenase (NADP+ dependent) 1-like | 2.03 | 2.10E-04 |
| *Pfkfb3* | ENSMUSG00000026773 | 6-phosphofructo-2-kinase/fructose-2,6-biphosphatase 3 | 2.12 | 6.10E-09 |
| *Gsdmc3* | ENSMUSG00000055827 | gasdermin C3 | 2.16 | 5.20E-05 |
| *Gsdmc2* | ENSMUSG00000056293 | gasdermin C2 | 2.21 | 2.70E-04 |
| *Per3* | ENSMUSG00000028957 | period circadian clock 3 | 2.35 | 3.50E-10 |
| *Mchr1* | ENSMUSG00000050164 | melanin-concentrating hormone receptor 1 | 2.36 | 3.70E-03 |
| *Tef* | ENSMUSG00000022389 | thyrotroph embryonic factor | 2.42 | 0.00E+00 |
| *Tsc22d3* | ENSMUSG00000031431 | TSC22 domain family, member 3 | 2.43 | 0.00E+00 |
| *Usp2* | ENSMUSG00000032010 | ubiquitin specific peptidase 2 | 2.56 | 2.10E-06 |
| *Acsm3* | ENSMUSG00000030935 | acyl-CoA synthetase medium-chain family member 3 | 2.58 | 1.30E-03 |
| *Hlf* | ENSMUSG00000003949 | hepatic leukemia factor | 2.63 | 5.70E-10 |
| *Per2* | ENSMUSG00000055866 | period circadian clock 2 | 2.87 | 0.00E+00 |
| *Tubb2b* | ENSMUSG00000045136 | tubulin, beta 2B class IIB | 2.9 | 1.20E-06 |
| *Ttr* | ENSMUSG00000061808 | transthyretin | 2.99 | 4.20E-07 |
| *Tcap* | ENSMUSG00000007877 | titin-cap | 3.21 | 7.70E-04 |
| *Per1* | ENSMUSG00000020893 | period circadian clock 1 | 3.77 | 0.00E+00 |
| *Mapk10* | ENSMUSG00000046709 | mitogen-activated protein kinase 10 | 4.7 | 9.00E-03 |
| *Dbp* | ENSMUSG00000059824 | D site albumin promoter binding protein | 4.71 | 0.00E+00 |
| *Elavl2* | ENSMUSG00000008489 | ELAV (embryonic lethal, abnormal vision, Drosophila)-like 2 (Hu antigen B) | 5 | 5.30E-03 |
| *Ptprn* | ENSMUSG00000026204 | protein tyrosine phosphatase, receptor type, N | 5.5 | 8.70E-03 |
| *Rab3c* | ENSMUSG00000021700 | RAB3C, member RAS oncogene family | 5.58 | 2.10E-04 |
| *Ngfr* | ENSMUSG00000000120 | nerve growth factor receptor (TNFR superfamily, member 16) | 5.66 | 1.10E-03 |
| *Prph* | ENSMUSG00000023484 | peripherin | 5.67 | 1.30E-03 |
| *Gap43* | ENSMUSG00000047261 | growth associated protein 43 | 5.87 | 1.80E-03 |
| *Chgb* | ENSMUSG00000027350 | chromogranin B | 6 | 7.20E-03 |
| *Orm1* | ENSMUSG00000039196 | orosomucoid 1 | 6.09 | 1.30E-03 |

| | | | | |
|---|---|---|---|---|
| *Mt3* | ENSMUSG00000031760 | metallothionein 3 | 6.46 | 8.40E-04 |
| *Cnr1* | ENSMUSG00000044288 | cannabinoid receptor 1 (brain) | 7.21 | 8.30E-04 |
| *Nos1* | ENSMUSG00000029361 | nitric oxide synthase 1, neuronal | 7.73 | 7.10E-04 |
| *Syt4* | ENSMUSG00000024261 | synaptotagmin IV | 7.95 | 5.10E-03 |
| *Ptprn2* | ENSMUSG00000056553 | protein tyrosine phosphatase, receptor type, N polypeptide 2 | 7.97 | 7.70E-04 |
| *Eef1a2* | ENSMUSG00000016349 | eukaryotic translation elongation factor 1 alpha 2 | 8.02 | 1.00E-04 |
| *Mmp12* | ENSMUSG00000049723 | matrix metallopeptidase 12 | 8.03 | 2.50E-03 |
| *Pcsk1n* | ENSMUSG00000039278 | proprotein convertase subtilisin/kexin type 1 inhibitor | 8.33 | 3.20E-04 |
| *Ciart* | ENSMUSG00000038550 | circadian associated repressor of transcription | 8.37 | 0.00E+00 |
| *Slc5a7* | ENSMUSG00000023945 | solute carrier family 5 (choline transporter), member 7 | 8.51 | 1.80E-03 |
| *Mrap2* | ENSMUSG00000042761 | melanocortin 2 receptor accessory protein 2 | 8.59 | 3.80E-03 |
| *Stmn3* | ENSMUSG00000027581 | stathmin-like 3 | 8.82 | 7.10E-04 |
| *Nefl* | ENSMUSG00000022055 | neurofilament, light polypeptide | 8.91 | 2.10E-04 |
| *Snap25* | ENSMUSG00000027273 | synaptosomal-associated protein 25 | 8.93 | 9.20E-04 |
| *Ppp2r2c* | ENSMUSG00000029120 | protein phosphatase 2, regulatory subunit B, gamma | 9.11 | 3.30E-03 |
| *Vip* | ENSMUSG00000019772 | vasoactive intestinal polypeptide | 9.38 | 1.80E-03 |
| *Slc18a3* | ENSMUSG00000100241 | solute carrier family 18 (vesicular monoamine), member 3 | 9.44 | 7.10E-04 |
| *Rit2* | ENSMUSG00000057455 | Ras-like without CAAX 2 | 9.6 | 6.00E-03 |
| *Cartpt* | ENSMUSG00000021647 | CART prepropeptide | 9.68 | 2.30E-03 |
| *Vstm2l* | ENSMUSG00000037843 | V-set and transmembrane domain containing 2-like | 10.16 | 8.70E-03 |
| *Cplx1* | ENSMUSG00000033615 | complexin 1 | 10.18 | 5.10E-03 |
| *Htr3a* | ENSMUSG00000032269 | 5-hydroxytryptamine (serotonin) receptor 3A | 10.22 | 1.10E-04 |
| *Slc10a4* | ENSMUSG00000029219 | solute carrier family 10 (sodium/bile acid cotransporter family), member 4 | 10.32 | 1.80E-03 |
| *Kcnq2* | ENSMUSG00000016346 | potassium voltage-gated channel, subfamily Q, member 2 | 10.36 | 2.80E-03 |
| *Pirt* | ENSMUSG00000048070 | phosphoinositide-interacting regulator of transient receptor potential channels | 10.55 | 1.70E-03 |
| *Htr3b* | ENSMUSG00000008590 | 5-hydroxytryptamine (serotonin) receptor 3B | 10.69 | 1.50E-03 |
| *Vat1l* | ENSMUSG00000046844 | vesicle amine transport protein 1 like | 11.2 | 2.20E-05 |
| *Npy* | ENSMUSG00000029819 | neuropeptide Y | 11.83 | 7.40E-04 |
| *A730017C20Rik* | ENSMUSG00000050875 | RIKEN cDNA A730017C20 gene | 11.85 | 3.60E-04 |
| *Zcchc12* | ENSMUSG00000036699 | zinc finger, CCHC domain containing 12 | 11.94 | 7.30E-04 |
| *Tubb3* | ENSMUSG00000062380 | tubulin, beta 3 class III | 11.99 | 3.10E-06 |
| *Jph3* | ENSMUSG00000025318 | junctophilin 3 | 12.34 | 1.30E-03 |
| *Alb* | ENSMUSG00000029368 | albumin | 12.42 | 8.40E-04 |
| *Chrna3* | ENSMUSG00000032303 | cholinergic receptor, nicotinic, alpha polypeptide 3 | 12.58 | 7.50E-04 |
| *Pagr1a* | ENSMUSG00000030680 | PAXIP1 associated glutamate rich protein 1A | 13.03 | 5.50E-10 |
| *Chrnb4* | ENSMUSG00000035200 | cholinergic receptor, nicotinic, beta polypeptide 4 | 13.09 | 3.30E-03 |
| *Hpcal4* | ENSMUSG00000046093 | hippocalcin-like 4 | 13.22 | 3.60E-04 |
| *Th* | ENSMUSG00000000214 | tyrosine hydroxylase | 13.4 | 4.40E-03 |
| *Fstl5* | ENSMUSG00000034098 | follistatin-like 5 | 13.81 | 2.50E-03 |
| *Dbh* | ENSMUSG00000000889 | dopamine beta hydroxylase | 14.24 | 2.20E-04 |
| *Ctnna2* | ENSMUSG00000063063 | catenin (cadherin associated protein), alpha 2 | 14.3 | 1.10E-03 |
| *Iglc2* | ENSMUSG00000076937 | immunoglobulin lambda constant 2 | 14.35 | 6.00E-03 |
| *Unc80* | ENSMUSG00000055567 | unc-80, NALCN activator | 14.89 | 1.30E-03 |
| *Tlx2* | ENSMUSG00000068327 | T cell leukemia, homeobox 2 | 15.25 | 6.20E-03 |
| *Slc7a14* | ENSMUSG00000069072 | solute carrier family 7 (cationic amino acid transporter, y+ system), member 14 | 15.77 | 1.80E-04 |
| *Cacng5* | ENSMUSG00000040373 | calcium channel, voltage-dependent, gamma subunit 5 | 16.27 | 6.60E-03 |
| *Serpina3k* | ENSMUSG00000058207 | serine (or cysteine) peptidase inhibitor, clade A, member 3K | 19.95 | 1.90E-05 |
| *Igkv15-103* | ENSMUSG00000076523 | immunoglobulin kappa chain variable 15-103 | 20.65 | 2.40E-03 |
| *Gm21320* | ENSMUSG00000110439 | predicted gene, 21320 | 23.4 | 6.70E-06 |
| *Gria2* | ENSMUSG00000033981 | glutamate receptor, ionotropic, AMPA2 (alpha 2) | 25.48 | 1.40E-03 |
| *Svop* | ENSMUSG00000042078 | SV2 related protein | 32.84 | 6.90E-04 |
| *Iglv2* | ENSMUSG00000076940 | immunoglobulin lambda variable 2 | 60.24 | 1.90E-03 |
| *Ighv1-36* | ENSMUSG00000094051 | immunoglobulin heavy variable 1-36 | 159.03 | 6.30E-03 |
| *Igkv4-68* | ENSMUSG00000076549 | immunoglobulin kappa variable 4-68 | 159.55 | 3.80E-03 |
| *Mup7* | ENSMUSG00000073842 | major urinary protein 7 | 208.29 | 2.90E-03 |
| *Igkv1-122* | ENSMUSG00000095497 | immunoglobulin kappa chain variable 1-122 | 240.32 | 1.20E-03 |

**Table S3.** All KEGG patwhays with significant changes in expression.

| KEGG number | Pathway |
|---|---|
| Upregulated Pathways | |
| mmu00020 | Citrate cycle (TCA cycle) |
| mmu00100 | Steroid biosynthesis |
| mmu00190 | Oxidative phosphorylation |
| mmu00240 | Pyrimidine metabolism |
| mmu00270 | Cysteine and methionine metabolism |
| mmu00280 | Valine, leucine and isoleucine degradation |
| mmu00480 | Glutathione metabolism |
| mmu00620 | Pyruvate metabolism |
| mmu00770 | Pantothenate and CoA biosynthesis |
| mmu00900 | Terpenoid backbone biosynthesis |
| mmu01200 | Carbon metabolism |
| mmu01212 | Fatty acid metabolism |
| mmu03008 | Ribosome biogenesis in eukaryotes |
| mmu03010 | Ribosome |
| mmu03013 | RNA transport |
| mmu03040 | Spliceosome |
| mmu03050 | Proteasome |
| mmu04144 | Endocytosis |
| mmu04146 | Peroxisome |
| mmu04216 | Ferroptosis |
| mmu04530 | Tight junction |
| mmu04721 | Synaptic vesicle cycle |
| mmu04722 | Neurotrophin signaling pathway |
| mmu04964 | Proximal tubule bicarbonate reclamation |
| mmu05014 | Amyotrophic lateral sclerosis (ALS) |
| Downregulated Pathways | |
| mmu04060 | Cytokine-cytokine receptor interaction |
| mmu04512 | ECM-receptor interaction |
| mmu04640 | Hematopoietic cell lineage |
| mmu04974 | Protein digestion and absorption |
| mmu05150 | Staphylococcus aureus infection |
| Pathways with up and downregulation | |
| mmu00100 | Steroid biosynthesis |
| mmu00270 | Cysteine and methionine metabolism |
| mmu00280 | Valine, leucine and isoleucine degradation |
| mmu00900 | Terpenoid backbone biosynthesis |
| mmu01200 | Carbon metabolism |
| mmu01212 | Fatty acid metabolism |
| mmu03010 | Ribosome |
| mmu04020 | Calcium signaling pathway |
| mmu04144 | Endocytosis |
| mmu04145 | Phagosome |
| mmu04146 | Peroxisome |
| mmu04151 | PI3K-Akt signaling pathway |
| mmu04216 | Ferroptosis |
| mmu04360 | Axon guidance |
| mmu04371 | Apelin signaling pathway |
| mmu04514 | Cell adhesion molecules (CAMs) |
| mmu04520 | Adherens junction |

| | |
|---|---|
| mmu04662 | B cell receptor signaling pathway |
| mmu04666 | Fc gamma R-mediated phagocytosis |
| mmu04710 | Circadian rhythm |
| mmu04974 | Protein digestion and absorption |
| mmu05012 | Parkinson's disease |
| mmu05014 | Amyotrophic lateral sclerosis (ALS) |
| mmu05202 | Transcriptional misregulation in cancer |
| mmu05230 | Central carbon metabolism in cancer |

**Table S4.** Enriched Molecular Functions in GF bladders.

| GO Molecular Function | # (total number in reference genome) | # (total number of genes in our dataset with that annotation) | Fold Enrichment | P value | FDR p-value |
|---|---|---|---|---|---|
| acetylcholine receptor activity | 6 | 2 | 77.3 | 5.02E-04 | 1.38E-02 |
| neuropeptide hormone activity | 12 | 2 | 38.65 | 1.61E-03 | 3.85E-02 |
| binding | 5268 | 40 | 1.76 | 9.69E-05 | 4.65E-03 |
| ligand-gated ion channel activity | 131 | 7 | 12.39 | 2.21E-06 | 4.24E-04 |
| ion channel activity | 330 | 8 | 5.62 | 1.05E-04 | 4.03E-03 |
| transmembrane transporter activity | 827 | 13 | 3.65 | 6.14E-05 | 5.89E-03 |
| transporter activity | 957 | 13 | 3.15 | 2.56E-04 | 8.18E-03 |
| cation channel activity | 116 | 4 | 8 | 1.79E-03 | 3.82E-02 |
| sequence-specific DNA binding RNA polymerase II transcription factor activity | 229 | 7 | 7.09 | 7.17E-05 | 4.59E-03 |
| acetylcholine receptor activity | 6 | 2 | 77.3 | 5.02E-04 | 1.38E-02 |
| neuropeptide hormone activity | 12 | 2 | 38.65 | 1.61E-03 | 3.85E-02 |
| binding | 5268 | 40 | 1.76 | 9.69E-05 | 4.65E-03 |
| ligand-gated ion channel activity | 131 | 7 | 12.39 | 2.21E-06 | 4.24E-04 |
| ion channel activity | 330 | 8 | 5.62 | 1.05E-04 | 4.03E-03 |

**Table S5.** Cellular components, reactome pathways and protein class enriched in GF tissue.

| GO Cellular Component | # (total number in reference genome) | # (total number of genes in our dataset with that annotation) | Fold Enrichment | P value | FDR p-value |
|---|---|---|---|---|---|
| neuron projection | 251 | 9 | 8.31 | 1.82E-06 | 1.16E-04 |
| cell projection | 387 | 9 | 5.39 | 5.22E-05 | 1.67E-03 |
| synapse | 130 | 4 | 7.14 | 2.68E-03 | 3.43E-02 |
| plasma membrane | 1731 | 19 | 2.55 | 1.42E-04 | 3.03E-03 |
| membrane | 2384 | 21 | 2.04 | 1.37E-03 | |

| Reactome pathways | # (total number in reference genome) | # (total number of genes in our dataset with that annotation) | Fold Enrichment | P value | FDR p-value |
|---|---|---|---|---|---|
| PPARA activates gene expression | 4 | 2 | > 100 | 2.71E-04 | 4.55E-02 |
| Catecholamine biosynthesis | 4 | 2 | > 100 | 2.71E-04 | 4.09E-02 |
| BMAL1:CLOCK,NPAS2 activates circadian gene expression | 20 | 5 | 57.97 | 6.52E-08 | 9.87E-05 |
| Circadian Clock | 28 | 5 | 41.41 | 2.83E-07 | 1.43E-04 |
| Acetylcholine Neurotransmitter Release Cycle | 17 | 4 | 54.56 | 1.81E-06 | 5.47E-04 |
| Neurotransmitter Release Cycle | 47 | 4 | 19.74 | 6.83E-05 | 1.29E-02 |
| Transmission across Chemical Synapses | 194 | 8 | 9.56 | 2.60E-06 | 6.55E-04 |
| Neuronal System | 307 | 9 | 6.8 | 8.87E-06 | 1.92E-03 |

| Protein Class | # (total number in reference genome) | # (total number of genes in our dataset with that annotation) | Fold Enrichment | P value | FDR p-value |
|---|---|---|---|---|---|
| acetylcholine receptor | 42 | 4 | 22.09 | 4.54E-05 | 9.75E-03 |
| GABA receptor | 42 | 4 | 22.09 | 4.54E-05 | 4.88E-03 |

**Table S6.** Shapiro-Wilk test for normal distribution.

| | SPF Male | GF Male | SPF Female | GF Female | SPF Male | GF Male | SPF male | Antibiotics male |
|---|---|---|---|---|---|---|---|---|
| **W** | 0.9712 | 0.9526 | 0.9251 | 0.7017 | 0.9675 | 0.8613 | 0.9382 | 0.8499 |
| **P value** | 0.9001 | 0.7615 | 0.5427 | 0.0064 | 0.8258 | 0.2331 | 0.5327 | 0.0284 |
| **Passed normality test (alpha=0.05)?** | **Yes** | **Yes** | **Yes** | **No** | **Yes** | **Yes** | **Yes** | **No** |
| **P value summary** | ns | ns | ns | ** | ns | ns | ns | * |

**Table S7.** Expression of genes for pattern recognition receptors.

| Gene symbol | Identifier | Gene name | Mean Total Counts[a] | Fold change | FDR p-value |
|---|---|---|---|---|---|
| Bcl10 | ENSMUSG00000028191 | B cell leukemia/lymphoma 10 | 1272 | 1.04 | 1E+00 |
| Card9 | ENSMUSG00000026928 | caspase recruitment domain family, member 9 | 53 | -1.42 | 1E+00 |
| Cd209a | ENSMUSG00000031494 | CD209a antigen | 498 | -1.36 | 1E+00 |
| Clec7a | ENSMUSG00000079293 | C-type lectin domain family 7, member a | 326 | -1.34 | 1E+00 |
| Havcr2 | ENSMUSG00000020399 | hepatitis A virus cellular receptor 2 | 41 | 1.00 | 1E+00 |
| Lgals1 | ENSMUSG00000068220 | lectin, galactose binding, soluble 1 | 14902 | -1.00 | 1E+00 |
| Ly75 | ENSMUSG00000026980 | lymphocyte antigen 75 | 131 | 1.16 | 1E+00 |
| Malt1 | ENSMUSG00000032688 | MALT1 paracaspase | 938 | -1.16 | 1E+00 |
| Mrc1 | ENSMUSG00000026712 | mannose receptor, C type 1 | 3268 | -1.43 | 1E-01 |
| Mrc2 | ENSMUSG00000020695 | mannose receptor, C type 2 | 2458 | -1.28 | 1E+00 |
| Nlrp3 | ENSMUSG00000032691 | NLR family, pyrin domain containing 3 | 53 | -1.34 | 1E+00 |
| Nod1 | ENSMUSG00000038058 | nucleotide-binding oligomerization domain containing 1 | 1133 | -1.16 | 1E+00 |
| Nod2 | ENSMUSG00000055994 | nucleotide-binding oligomerization domain containing 2 | 63 | 1.23 | 1E+00 |
| Src | ENSMUSG00000027646 | Rous sarcoma oncogene | 6555 | 1.01 | 1E+00 |
| Syk | ENSMUSG00000021457 | spleen tyrosine kinase | 638 | -1.14 | 1E+00 |
| Tlr1 | ENSMUSG00000044827 | toll-like receptor 1 | 85 | -1.55 | 1E+00 |
| Tlr2 | ENSMUSG00000027995 | toll-like receptor 2 | 588 | -1.17 | 1E+00 |
| Tlr3 | ENSMUSG00000031639 | toll-like receptor 3 | 1187 | -1.02 | 1E+00 |
| Tlr4 | ENSMUSG00000039005 | toll-like receptor 4 | 955 | -1.19 | 1E+00 |
| Tlr5 | ENSMUSG00000079164 | toll-like receptor 5 | 226 | -1.25 | 1E+00 |
| Tlr6 | ENSMUSG00000051498 | toll-like receptor 6 | 65 | -1.11 | 1E+00 |
| Tlr7 | ENSMUSG00000044583 | toll-like receptor 7 | 238 | -1.03 | 1E+00 |
| Tlr8 | ENSMUSG00000040522 | toll-like receptor 8 | 108 | -1.21 | 1E+00 |
| Tlr9 | ENSMUSG00000045322 | toll-like receptor 9 | 112 | -1.22 | 1E+00 |
| Trem1 | ENSMUSG00000042265 | triggering receptor expressed on myeloid cells 1 | 1 | -1.87 | 1E+00 |
| Tyrobp | ENSMUSG00000030579 | TYRO protein tyrosine kinase binding protein | 1129 | -1.22 | 1E+00 |

[a]Mean Total Counts is the mean of a normalized number of reads for each gene in the three SPF samples.

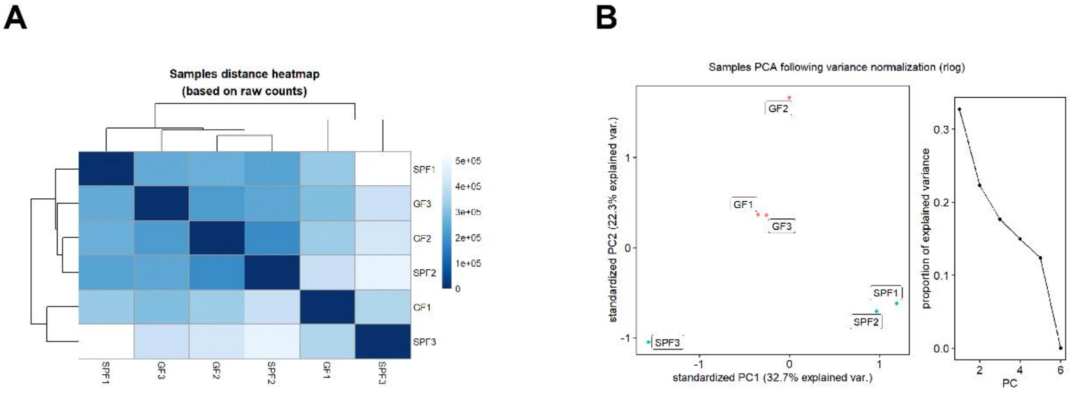


**Figure S1.** RNA-Seq sample comparison of GF and SPF groups. A. Complete clustering of samples using Euclidean distance calculated on raw counts B.PCA on rlog variance normalized counts.

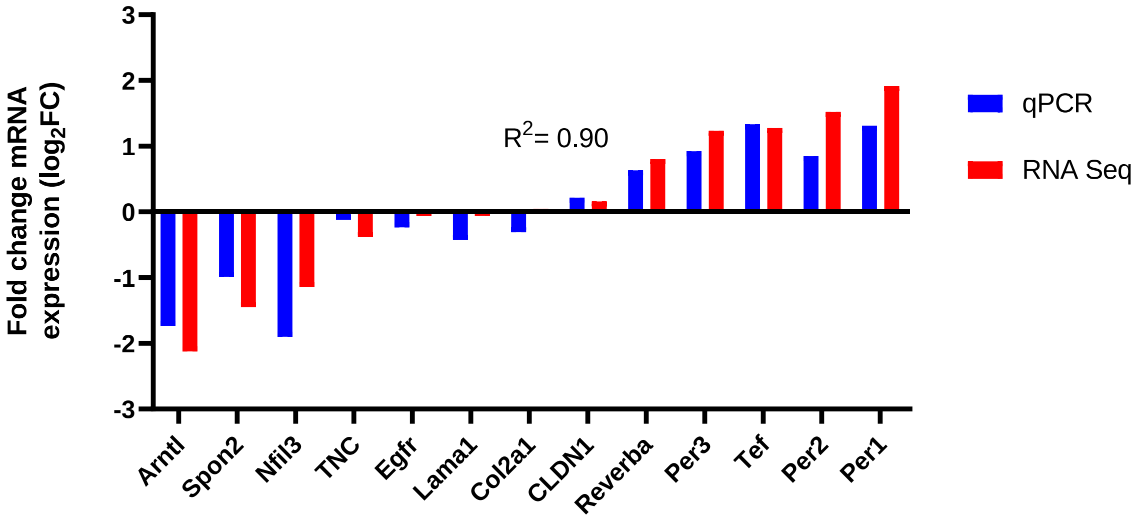


**Figure S2.** Concurrence of log2(fold change) from qPCR and RNA-seq analysis for 13 exemplar genes, correlation expressed with R2 correlation coefficient; Bars represent mean value.

**A**

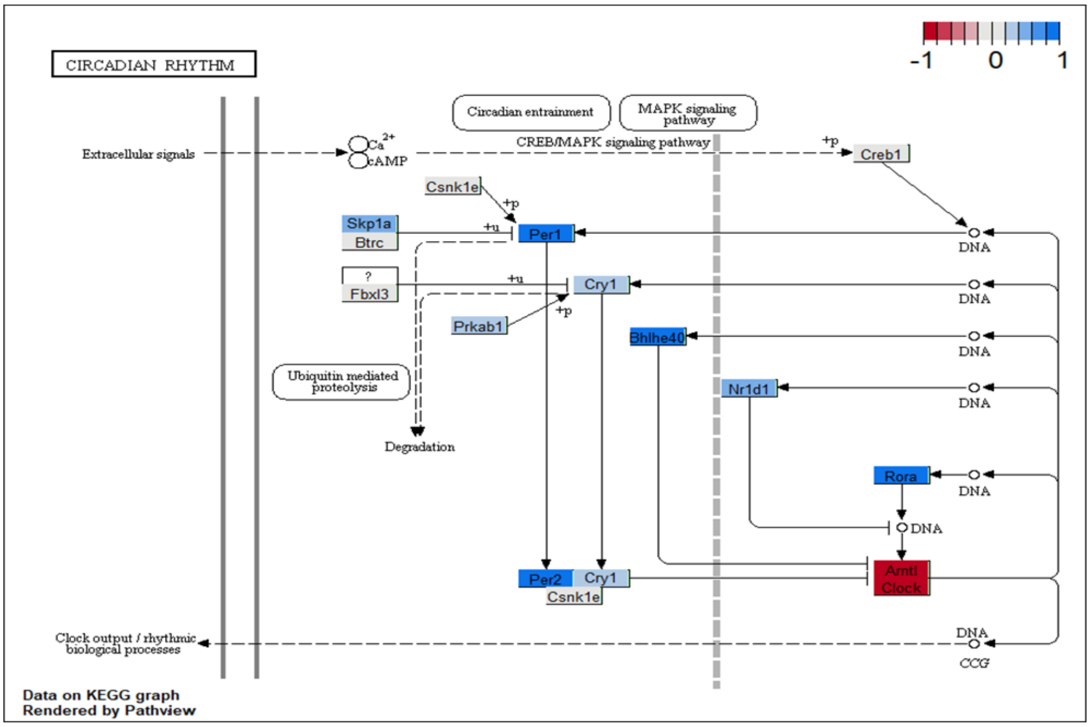
-1
0
1
CIRCADIAN RHYTHM
Circadian entrainment
MAPK signaling pathway
Extracellular signals
Ca2+
cAMP
CREB/MAPK signaling pathway
+p
Creb1
Csnk1e
+p
Skp1a
Btrc
+u
Per1
DNA
?
Fbxl3
+u
Cry1
DNA
Prkab1
+p
Bhlhe40
DNA
Ubiquitin mediated proteolysis
Nr1d1
DNA
Degradation
Rora
DNA
DNA
Per2
Cry1
Csnk1e
Arntl
Clock
Clock output / rhythmic biological processes
DNA
CCG
Data on KEGG graph
Rendered by Pathview

**B**

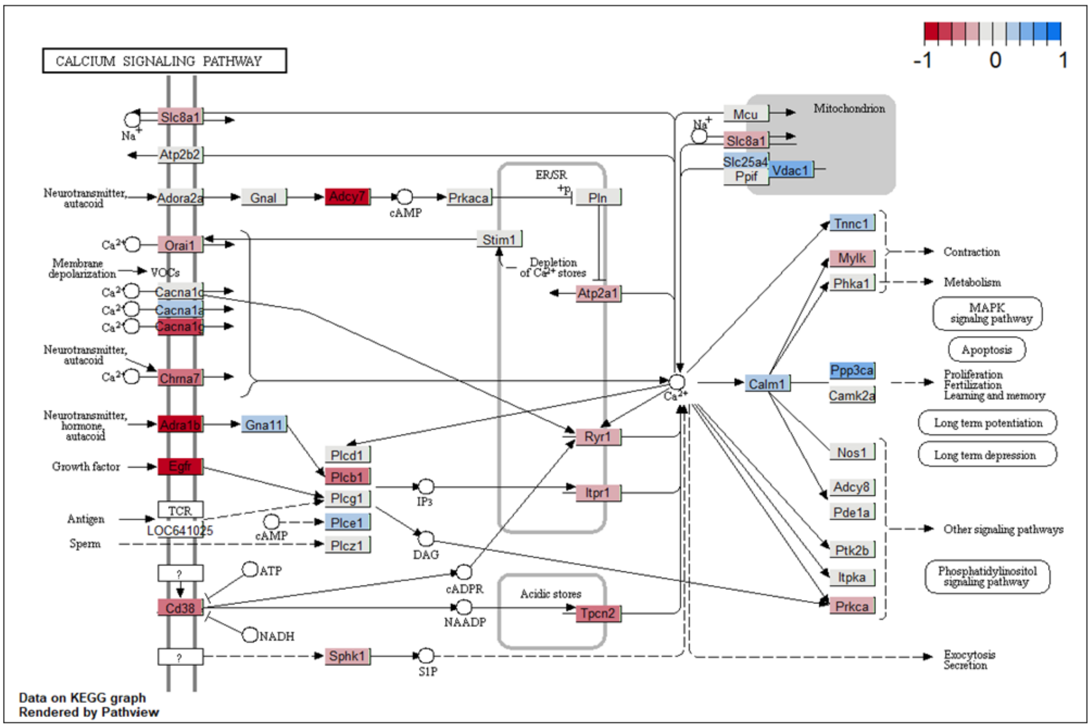
-1
0
1
CALCIUM SIGNALING PATHWAY
Slc8a1
Na+
Atp2b2
Mcu
Na+
Slc8a1
Mitochondrion
Slc25a4
Ppif
Vdac1
ER/SR
Neurotransmitter, autacoid
Adora2a
Gnal
Adcy7
cAMP
Prkaca
+p
Pln
Orai1
Stim1
Tnnc1
Mylk
Contraction
Phka1
Metabolism
Membrane depolarization
VOCs
Cacna1c
Cacna1a
Cacna1g
Depletion of Ca2+ stores
Atp2a1
MAPK signaling pathway
Apoptosis
Neurotransmitter, autacoid
Chrna7
Ca2+
Calm1
Ppp3ca
Camk2a
Proliferation
Fertilization
Learning and memory
Long term potentiation
Long term depression
Neurotransmitter, hormone, autacoid
Adra1b
Gna11
Ryr1
Plcd1
Nos1
Growth factor
Egfr
Plcb1
Adcy8
Plcg1
IP3
Itpr1
Pde1a
Antigen
TCR
LOC641025
cAMP
Plce1
Other signaling pathways
Sperm
Plcz1
DAG
Ptk2b
?
ATP
cADPR
Itpka
Phosphatidylinositol signaling pathway
Acidic stores
Cd38
NAADP
Tpcn2
Prkca
NADH
?
Sphk1
S1P
Exocytosis
Secretion
Data on KEGG graph
Rendered by Pathview

C

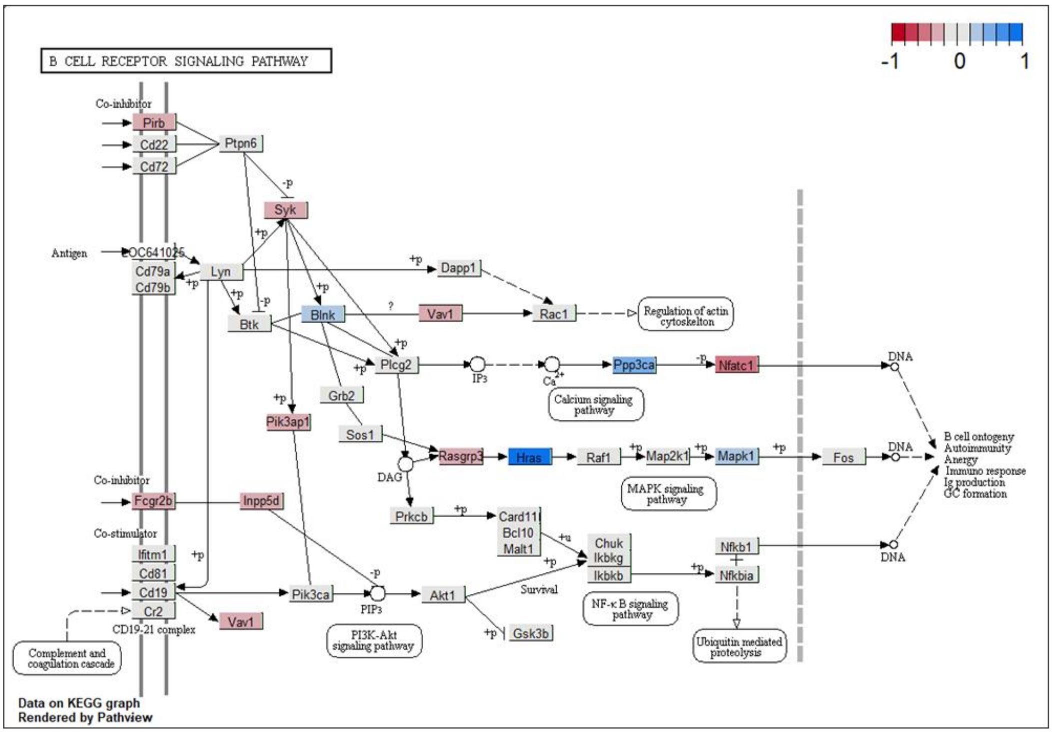


**Figure S3.** KEGG pathways with significant change in gene expression. The colouring is rendered from the summary $log_2$(fold change) of all genes in one gene node, representing multiple genes with a redundant functional role. **A.** Circadian rhythm **B.** Calcium signalling pathway **C.** B cell receptor signalling pathway.

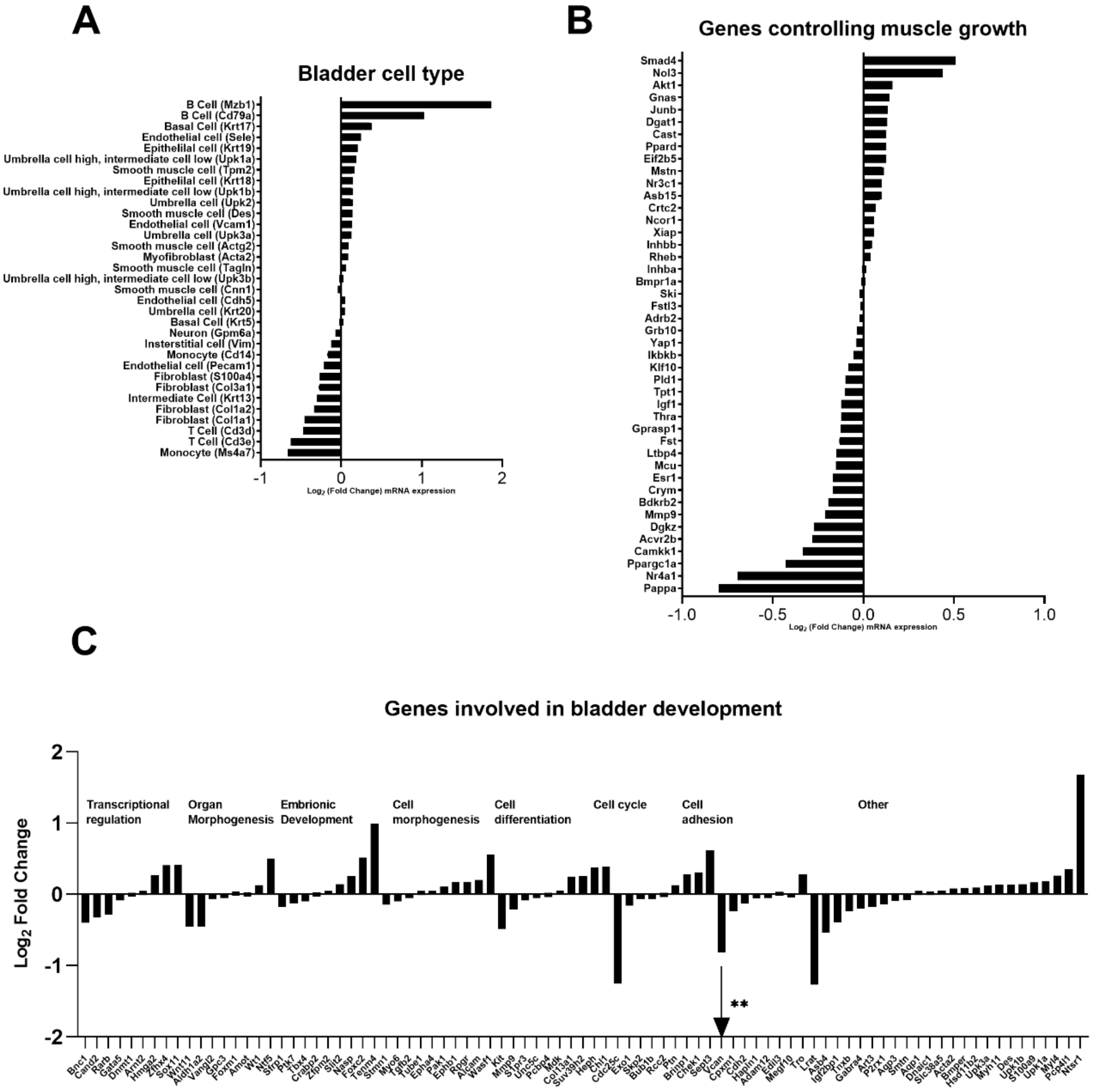


**Figure S4.** Log2 Fold change (GF vs SPF) of gene expression for **A.** gene markers for bladder cell types **B.** genes controlling muscle growth **C.** genes involved in bladder development; arrow with asterisks shows significant change (FDR p<0.01)
